# Urban relatives ameliorate survival disparities for genitourinary cancer in rural patients

**DOI:** 10.1002/cam4.7058

**Published:** 2024-03-13

**Authors:** Mouneeb Choudry, Kassandra Dindinger‐Hill, Jacob Ambrose, Joshua Horns, Jeffrey Vehawn, Hailie Gill, Nicole Z. Murray, Trevor E. Hunt, Christopher Martin, Benjamin Haaland, Jonathan Chipman, Heidi A. Hanson, Brock B. O'Neil

**Affiliations:** ^1^ Huntsman Cancer Institute University of Utah Salt Lake City Utah USA; ^2^ Department of Urology Mayo Clinic Phoenix Arizona USA; ^3^ Department of Urology University of Rochester Rochester New York USA; ^4^ Division of Urology University of Utah Salt Lake City Utah USA; ^5^ Computational Sciences and Engineering Division Oak Ridge National Laboratory Oak Ridge Tennessee USA

**Keywords:** family, rural health, urogenital neoplasms

## Abstract

**Introduction:**

Patients living in rural areas have worse cancer‐specific outcomes. This study examines the effect of family‐based social capital on genitourinary cancer survival. We hypothesized that rural patients with urban relatives have improved survival relative to rural patients without urban family.

**Methods:**

We examined rural and urban based Utah individuals diagnosed with genitourinary cancers between 1968 and 2018. Familial networks were determined using the Utah Population Database. Patients and relatives were classified as rural or urban based on 2010 rural–urban commuting area codes. Overall survival was analyzed using Cox proportional hazards models.

**Results:**

We identified 24,746 patients with genitourinary cancer with a median follow‐up of 8.72 years. Rural cancer patients without an urban relative had the worst outcomes with cancer‐specific survival hazard ratios (HRs) at 5 and 10 years of 1.33 (95% CI 1.10–1.62) and 1.46 (95% CI 1.24–1.73), respectively relative to urban patients. Rural patients with urban first‐degree relatives had improved survival with 5‐ and 10‐year survival HRs of 1.21 (95% CI 1.06–1.40) and 1.16 (95% CI 1.03–1.31), respectively.

**Conclusions:**

Our findings suggest rural patients who have been diagnosed with a genitourinary cancer have improved survival when having relatives in urban centers relative to rural patients without urban relatives. Further research is needed to better understand the mechanisms through which having an urban family member contributes to improved cancer outcomes for rural patients. Better characterization of this affect may help inform policies to reduce urban–rural cancer disparities.

## Graphical abstract

Patients living in rural areas have worse overall survival and cancer‐specific outcomes. This study examines the effect of family‐based social capital on genitourinary cancer survival. We hypothesized that rural patients with urban relatives have improved survival relative to rural patients without urban family.

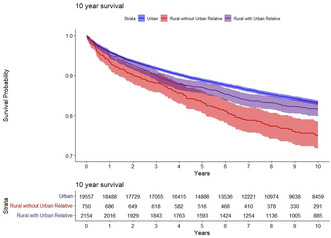

## 1 INTRODUCTION

Significant cancer care disparities exist between rural and urban patients.^1^, ^2^, ^3^ Patients in rural areas have been shown to experience a higher incidence of preventable cancers, are more likely to receive a diagnosis at a later stage and have higher cancer‐specific mortality than patients living in urban areas.^1^, ^4^ Several possible explanations have been explored, though none have completely accounted for the breadth of the disparity.

Screening and preventive care rates have been shown to be lower for patients living in rural areas, however rural patients who receive similar screening and treatment to urban patients have similar outcomes.^5^ Identifying policies that are effective at achieving similar screening and treatment rates among rural patients is challenging.^6^, ^7^, ^8^ Rural cancer patients are also more likely to have lower economic and social capital than urban patients. This can result in barriers to care including financial limitations, transportation difficulties, and lack of insurance coverage.^9^

Studies have found that social support is an important aspect of cancer care in rural patients.^10^ The type of social support that is most beneficial to rural patients has not been well explored. In this study, we seek to better understand how rural patients with relatives in urban areas who live nearer to cancer centers and cancer resources may contribute to better outcomes for rural patients.

We leveraged a unique data resource, the Utah Population Database (UPDB), that contains information about cancer, healthcare utilization, and residential histories for both patients and their family members to better characterize the impact of rural patients with urban relatives. We compared cancer outcomes between three groups of cancer patients: (a) rural without urban relatives, (b) rural with urban relatives, and (c) urban. We hypothesized that rural patients who have family members in urban areas have better overall survival (OS) compared to rural patients who lack relatives in urban areas.

## 2 MATERIALS AND METHODS

### 2.1 Study design

We performed a retrospective cohort analysis of adults diagnosed with genitourinary cancers, including: prostate, bladder, kidney, penile, and testicular cancers between 1990 and 2018. Due to potential concerns about privacy and misuse of this unique data resource, access to UPDB by researchers requires selection of specific cancer subsets from the entire data. We selected the included genitourinary cancers both because of our research group's expertise and to represent a variety of cancer types that affect different populations, incidences, affected age groups, and mortality risk.

### 2.2 Data setting

Patients were classified as living in either rural or urban areas based on the 2010 rural–urban commuting area (RUCA) codes associated with their zip code of residence at the time of cancer diagnosis.^11^ RUCA codes include whole numbers ranging from 1 to 10, with 1 indicating a metropolitan area core with primary flow within an urbanized area and 10 indicating rural areas with primary flow to tracts outside of urbanized areas or urban clusters. Whole integers between these two extremes represent a range of metropolitan, micropolitan, and small towns with various flows. Secondary numbers range from 1.10 to 10.3 and further breakdown whole integer categories by secondary flows to different urbanized areas and urban clusters. If a patient lived in an area classified by RUCA codes 4, 5, 7, 8, 10, 10.3, they were considered rural for this study.

Adult first‐ and second‐degree relatives were identified and classified as urban or rural based on the zip code or county of residence at the time of the patients' diagnosis or, when unavailable, the county or zip code of residence before or after diagnosis. Family members were classified as living in an urban area if their residence at the time of patient diagnosis was classified by RUCA codes 1, 2, 3, 4.1, and 7.1.

### 2.3 Statistical analysis

All statistical analyses were performed using R software, with a *p* value <0.05 denoting statistical significance. OS was analyzed using Cox proportional hazards models after adjusting for sex, age, race, cancer type, SEER cancer stage, year of cancer diagnosis, health improvement index (HII),^12^ and Simpson's diversity index.^13^ OS hazard ratios (HR) were then used to calculate the probability of survival for patients grouped as urban, rural without urban relatives, and rural with urban relatives. The results were then plotted as Kaplan–Meier curves.

Sensitivity analyses further examined the robustness of results by exploring whether subjects with larger family networks had a different outcome to account for the potential explanatory effect of having larger families. These were demonstrated in the form of OS. Patients diagnosed with genitourinary cancer living within urban regions described by the RUCA were utilized as the reference group.

### 2.4 Main outcomes

There were minimal differences between groups when evaluating any number of first‐degree relatives versus those with a minimum of five first‐degree relatives (Table S1). We also stratified cancers into subgroups, including prostate and non‐prostate GU cancer, finding minimal differences between groups (Table S2).

## 3 RESULTS

We identified 22,766 individuals diagnosed with genitourinary cancer between 1990 and 2018 in the UPDB. Median follow‐up was 8.78 years. The population predominantly represented men (81%) and white individuals (86.7%). Most patients had prostate cancer, which accounted for 71.9% of all the studied cancers (Table 1).

**TABLE 1:** Baseline patient demographics stratified by rural or urban status.

	Overall	Urban	Rural with Urban Relative	Rural without Urban Relative	*p*‐value
Total Patients	22,766 (100%)	19,808 (87.0%)	2189 (9.6%)	769 (3.4%)	
Age					*p* = <0.001
55 or younger	3894 (17.1%)	3444 (17.4%)	286 (13.1%)	164 (21.3%)	
56–64	6207 (27.3%)	5416 (27.3%)	595 (27.2%)	196 (25.5%)	
65–74	7827 (34.4%)	6771 (34.2%)	803 (36.7%)	253 (32.9%)	
75 or older	4838 (21.3%)	4177 (21.1%)	505 (23.1%)	156 (20.3%)	
Sex					*p* = 0.024
Male	20,920 (91.9%)	18,198 (91.9%)	2032 (92.8%)	690 (89.7%)	
Female	1846 (8.1%)	1610 (8.1%)	157 (7.2%)	79 (10.3%)	
Race					*p* = 0.017
White	22,020 (96.7%)	19,193 (96.9%)	2105 (96.2%)	722 (93.9%)	
Asian	46 (0.2%)	46 (0.2%)	0 (0%)	0 (0%)	
Black or African American	35 (0.2%)	35 (0.2%)	0 (0%)	0 (0%)	
Native Hawaiian or Pacific Islander	10 (0.04%)	10 (0.1%)	0 (0%)	0 (0%)	
American Indian or Alaska Native	23 (0.1%)	4 (0.02%)	6 (0.3%)	13 (1.7%)	
Multiple Races	632 (2.8%)	520 (2.6%)	78 (3.6%)	34 (4.4%)	
Median Follow‐up, years (Std. Dev.)	8.8 (5.1–13.3)	8.9 (5.1–13.3)	8.4 (4.8–12.9)	7.9 (4.3–12.8)	*p* = <0.001
Cancer Type					*p* = <0.001
Prostate	16,364 (71.9%)	14,212 (71.8%)	1640 (74.9%)	512 (66.6%)	
Bladder	2775 (12.2%)	2403 (12.1%)	262 (12.0%)	110 (14.3%)	
Kidney	2416 (10.6%)	2108 (10.6%)	217 (9.9%)	91 (11.8%)	
Testis	947 (4.2%)	857 (4.3%)	51 (2.3%)	39 (5.1%)	
Other	264 (1.2%)	228 (1.2%)	19 (0.9%)	17 (2.2%)	
Year of Cancer Diagnosis					*p* = <0.001
<2000	4835 (21.2%)	4228 (21.3%)	389 (17.8%)	218 (28.4%)	
2000–2004	4268 (18.8%)	3704 (18.7%)	410 (18.7%)	154 (20.0%)	
2005–2009	5892 (25.9%)	5113 (25.8%)	602 (27.5%)	177 (23.0%)	
2010–2014	6362 (28.0%)	5539 (28.0%)	645 (29.5%)	178 (23.2%)	
2015–2018	1224 (6.2%)	143 (6.5%)	42 (5.5%)	1409 (6.2%)	
Health Improvement Index					*p* = <0.001
Very Low	4302 (18.9%)	4302 (21.7%)	0 (0%)	0 (0%)	
Low	5175 (22.7%)	4549 (23.0%)	499 (22.8%)	127 (16.5%)	
Average	5205 (22.9%)	4104 (20.7%)	795 (36.3%)	306 (39.8%)	
High	4679 (20.6%)	3890 (19.6%)	587 (26.8%)	202 (26.3%)	
Very High	3405 (15.0%)	2963 (15.0%)	308 (14.1%)	134 (17.4%)	
Simpson's Diversity Index	0.23 (0.17–0.35)	0.23 (0.18–0.35)	0.19 (0.12–0.26)	0.19 (0.14–0.26)	*p* = <0.001
5 year OS					*p* = <0.001
Alive	18,346 (80.6%)	16,073 (81.1%)	1715 (78.3%)	558 (72.6%)	
Dead	4420 (19.4%)	3735 (18.9%)	474 (21.7%)	211 (27.4%)	
10 year OS					*p* = <0.001
Alive	15,747 (69.2%)	13,816 (69.7%)	1477 (67.5%)	454 (59.0%)	
Dead	7019 (30.8%)	5992 (30.3%)	712 (32.5%)	315 (41.0%)	

Abbreviation: OS, overall survival.

**FIGURE 1:**
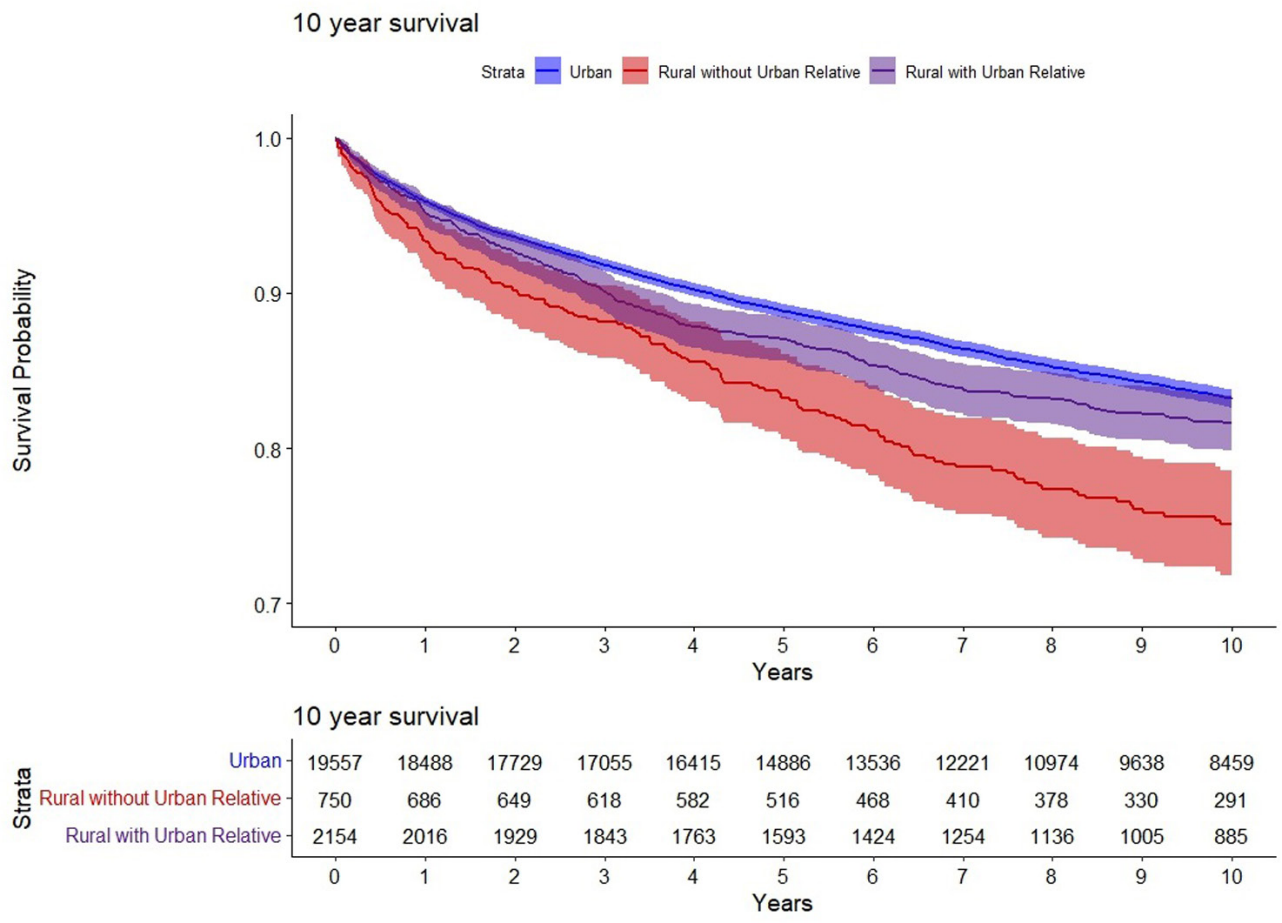
Overall‐specific survival.

We found urban patients (reference group) had a significantly higher OS at 5 and 10 years compared to rural patients with and without an urban first‐degree relative **(**Figure 1 **)**. Rural patients without an urban first‐degree relative had a 41% and 46% greater mortality risk than urban patients, as seen with OS HRs at 5 and 10 years (5‐year HR 1.41, 95% CI 1.22–1.63, *p* < 0.001) and (10‐year HR 1.46, 95% CI 1.30–1.65, *p* < 0.001) respectively (Table 2). The significantly higher OS of urban patients compared to rural patients without urban relatives is further supported by higher cancer‐specific survival curves at both 5 and 10 year (Figures S1 and S2).

**TABLE 2:** Five‐ and ten‐year overall survival comparing rural patients with and without urban relatives to urban patients.

	5‐year OS	*p*‐value	10‐year OS	*p*‐value
	HR (95% CI)		HR (95% CI)	
Urban	REF		REF	
Rural with urban relatives	1.19 (1.07–1.32)	0.002	1.13 (1.04–1.23)	0.004
Rural without urban relatives	1.41 (1.22–1.63)	<0.001	1.46 (1.30–1.65)	<0.001

Abbreviation: OS, overall survival.

## 4 DISCUSSION

In this study, we confirmed work supported by other researchers that rural cancer patients have worse outcomes. However, we were able to take a deeper dive and show that familial ties also affect this relationship. Having urban first‐degree relatives appear to improve survival outcomes for rural patients.

There are several possible explanations for this observation. First, when patients have family members in urban areas, they may have greater family‐based social capital that facilitates care in urban centers. Families may be able to provide improved access to care via temporary lodging, transportation or other resources that facilitate care. This may help patients overcome common barriers to accessing high‐quality tertiary cancer care in rural patients.^14^ Second, having a family network near centralized cancer care may permit rural patients to have increased levels of social support, including caregiver support and knowledge about local high‐quality resources, needed to achieve better outcomes. Third, rural patients may have access to additional financial resources through family networks living in urban areas similar to immigrant populations who are able to share income with family that is abroad to help them attain a better living standard.

These potential explanations are supported by prior studies highlighting the impact of family and social support on access to care and patient health. Strong family support was shown to improve emotional and physical health in the postoperative period in patients diagnosed with digestive cancers.^15^ On the other hand, lower levels of family support for young breast cancer patients was significantly associated with cost‐related lack of access to care.^16^ Another study evaluating a collaborative care model to improve social support for disadvantaged patients, found improvement in depression severity when patients received enhanced social support.^17^ Our study is the first to specifically evaluate the impact of having an urban relative for patients physically living in rural areas. In the context of existing literature, it is a reasonable presumption that enhanced family support paired with enhanced social resources from urban relatives living physically nearer to cancer centers contributes to improved outcomes for rural patients. More research is needed to determine which characteristics of rural patients and urban relatives lead to the greatest improvements in survival outcomes.

Various policies and initiatives have attempted to narrow the gap in outcomes between rural and urban patients in recent years. Awareness of the issue and efforts to improve upon these disparities have recently hastened. The American Society of Clinical Oncology and the National Institute of Health have both targeted this issue with separate campaigns, taking unprecedented steps to address lagging quality of care and outcomes.^18^, ^19^ The results of our study suggest that policies to provide resources and support to rural patients may have real impacts on cancer outcomes. Possible interventions may include providing expanded insurance access, transportation and lodging, support for caregivers, or cash for cancer related care. Policies may further attempt to simulate familial networks by appointing case workers with access to resources to help patients overcome additional barriers that may arise from traveling in unfamiliar locations.

Our study has five main limitations. First, our data is limited by the retrospective nature of the study. Second, our study population consisted of patients residing in Utah, limiting generalizability to other states and more diverse populations. Third, due to the selection of genitourinary cancer patients, this cohort was predominantly male. Fourth, our database did not contain comorbidity data. Fifth, our database did not contain extensive information which would have allowed us to extrapolate the specific social supports that reduce mortality for rural cancer patients with urban relatives.

More research is warranted to include an increased percentage of female patients. Nevertheless, this study represents a novel and unique opportunity to understand the role of family in supporting patients with cancer to improve outcomes.

## 5 CONCLUSIONS

Individuals diagnosed with cancer who live in rural areas have worse survival as compared to their urban counterparts, but this relationship appears to be improved by the presence of family who live in urban areas. Further research is needed to better understand the mechanisms through which having an urban family member may contribute to improved cancer outcomes for rural patients.

## AUTHOR CONTRIBUTIONS

**Mouneeb Choudry:** Conceptualization (lead); methodology (lead); writing – original draft (lead). **Kassandra Dindinger‐Hill:** Writing – review and editing (supporting). **Jacob Ambrose:** Formal analysis (equal); writing – review and editing (supporting). **Jeffrey Vehawn:** Formal analysis (equal); writing – review and editing (supporting). **Hailie Gill:** Writing – review and editing (supporting). **Nicole Z. Murray:** Writing – review and editing (supporting). **Trevor E. Hunt:** Writing – review and editing (supporting). **Christopher Martin:** Writing – review and editing (supporting). **Joshua Horns:** Formal analysis (equal); writing – review and editing (supporting). **Benjamin Haaland:** Formal analysis (equal); writing – review and editing (supporting). **Jonathan Chipman:** Formal analysis (equal); writing – review and editing (supporting). **Heidi A. Hanson:** Writing – review and editing (supporting). **Brock B. O'Neil:** Writing – review and editing (supporting).

## FUNDING INFORMATION

National Institute of Health [NIH].

## PRÉCIS

Rural patients who have been diagnosed with a genitourinary cancer have improved survival when having relatives in urban centers relative to rural patients without urban relatives.

## CONFLICT OF INTEREST STATEMENT

This content is solely the responsibility of the authors and does not necessarily represent the official views of the National Institutes of Health.

## ETHICS STATEMENT

Ethics approval and informed consent was waived for this study by The University of Utah Institutional Review Board, as the data obtained for this research was acquired via a non‐consent based cancer registry.

## Supporting information


Figure S1.
Figure 2.

Table S1.
Table S2.

## Data Availability

Data from this study is unable to be made publicly available because the database used for this work is not made freely available to the public.

## References

[1] Blake KD , Moss JL , Gaysynsky A , Srinivasan S , Croyle RT . Making the case for investment in rural cancer control: an analysis of rural cancer incidence, mortality, and funding trends. Cancer Epidemiol Biomarkers Prev. 2017;26(7):992‐997. doi:10.1158/1055-9965.EPI-17-0092 28600296 PMC5500425

[2] Singh GK . Rural‐urban trends and patterns in cervical cancer mortality, incidence, stage, and survival in the United States, 1950‐2008. J Community Health. 2012;37(1):217‐223. doi:10.1007/s10900-011-9439-6 21773819

[3] Weaver KE , Geiger AM , Lu L , Case LD . Rural–urban disparities in health status among US cancer survivors. Cancer. 2013;119(5):1050‐1057. doi:10.1002/cncr.27840 23096263 PMC3679645

[4] Zahnd WE , James AS , Jenkins WD , et al. Rural–urban differences in cancer incidence and trends in the United States. Cancer Epidemiol Biomarkers Prev. 2018;27(11):1265‐1274. doi:10.1158/1055-9965.EPI-17-0430 28751476 PMC5787045

[5] Bennett KJ , Probst JC , Bellinger JD . Receipt of cancer screening services: surprising results for some rural minorities. J Rural Health. 2012;28(1):63‐72. doi:10.1111/j.1748-0361.2011.00365.x 22236316

[6] Unger JM , Moseley A , Symington B , Chavez‐Macgregor M , Ramsey SD , Hershman DL . Geographic distribution and survival outcomes for rural patients with cancer treated in clinical trials. JAMA Netw Open. 2018;1(4):e181235. doi:10.1001/JAMANETWORKOPEN.2018.1235 30646114 PMC6324281

[7] Meilleur A , Subramanian SV , Plascak JJ , Fisher JL , Paskett ED , Lamont EB . Rural Residence and Cancer Outcomes in the United States: Issues and Challenges. Cancer Epidemiol Biomarkers Prev. 2013;22:1657‐1667. doi:10.1158/1055-9965.EPI-13-0404 24097195 PMC3814162

[8] Yabroff KR , Han X , Zhao J , Nogueira L , Jemal A . Rural Cancer Disparities in the United States: A Multilevel Framework to Improve Access to Care and Patient Outcomes. JCO Oncol Pract. 2020;16(7):409‐413. doi:10.1200/OP.20.00352 32574130

[9] Petermann V , Zahnd WE , Vanderpool RC , et al. How cancer programs identify and address the financial burdens of rural cancer patients. Support Care Cancer. 2022;30(3):2047‐2058. doi:10.1007/s00520-021-06577-z 34655327 PMC9380718

[10] Koopman C , Angell K , Turner‐Cobb JM , et al. Distress, coping, and social support among rural women recently diagnosed with primary. Breast Cancer. 2001;7:25‐33. 10.1046/j.1524-4741.2001.007001025.x 11348412

[11] Economic Research Service . Rural‐Urban Commuting Area (RUCA) Codes . 2010. Accessed January 15s, 2023. https://www.ers.usda.gov/data‐products/rural‐urban‐commuting‐area‐codes/documentation/

[12] Utah Department of Health . *The Utah Health Improvement Index*.; 2018. Accessed April 7, 2022 https://health.utah.gov/disparities/data/ohd/UtahHII.pdf

[13] Royal Geographical Society . A Guide to Simpson's Diversity Index . Accessed April 7, 2022. https://www.rgs.org/CMSPages/GetFile.aspx?nodeguid=018f17c3‐a1af‐4c72‐abf2‐4cb0614da9f8&lang=en‐GB

[14] Douthit N , Kiv S , Dwolatzky T , Biswas S . Exposing some important barriers to health care access in the rural USA. Public Health. 2015;129(6):611‐620. doi:10.1016/j.puhe.2015.04.001 26025176

[15] Wang MM , Chen DM , Zhang O , et al. Effect of family support on quality of postoperative life in patients with digestive cancer. Ann Palliat Med. 2020;9(4):2072‐2078. doi:10.21037/apm-20-1129 32648467

[16] Katapodi MC , Ellis KR , Schmidt F , Nikolaidis C , Northouse LL . Predictors and interdependence of family support in a random sample of long‐term young breast cancer survivors and their biological relatives. Cancer Med. 2018;7(10):4980‐4992. doi:10.1002/cam4.1766 30187678 PMC6198202

[17] Steinman LE , Gasca A , Hoeft TJ , et al. "we are the sun for our community:" partnering with community health workers/promotores to adapt, deliver and evaluate a home‐based collaborative care model to improve equity in access to quality depression care for older U.S. Latino adults who are underserved. Front Public Health. 2023;11:1079319. doi:10.3389/fpubh.2023.1079319 36817932 PMC9932325

[18] Kennedy AE , Vanderpool RC , Croyle RT , Srinivasan S . An overview of the national cancer institute's initiatives to accelerate rural cancer control research. Cancer Epidemiol Biomarkers Prev. 2018;27(11):1240‐1244. doi:10.1158/1055-9965.EPI-18-0934 30385495 PMC13577081

[19] Cavallo J . ASCO launches task force to address the cancer care gap in rural America. The ASCO Post.

