# Correction to “urban relatives ameliorate survival disparities for genitourinary cancer in rural patients”

**DOI:** 10.1002/cam4.7153

**Published:** 2024-04-01

Choudry M, Dindinger‐Hill K, Ambrose J, et al. Urban relatives ameliorate survival disparities for genitourinary cancer in rural patients. *Cancer Med*. 2024;13:e7058. https://doi.org/10.1002/cam4.7058.

The revised co‐author name which is shown below has been added to the online version of the article.

Trevor C. Hunt.

