# Supplementary material for: Urban relatives ameliorate survival disparities for genitourinary cancer in rural patients
Source: Cancer Med. 2024 Mar 13;13(5):e7058. doi: 10.1002/cam4.7058 (PMC10935886; doi:10.1002/cam4.7058)
Supplement: Supplementary file 1 — Figure S1. Figure 2. [file CAM4-13-e7058-s002.docx]

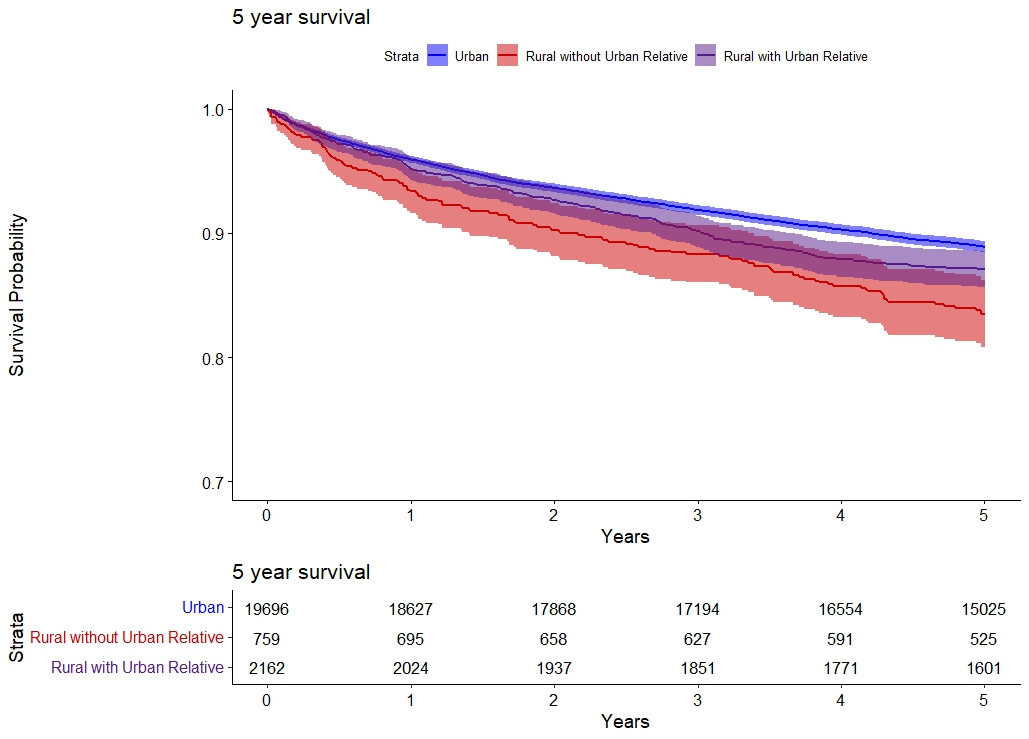


Supplementary Figure 1. Five Year Cancer Specific Survival Curve


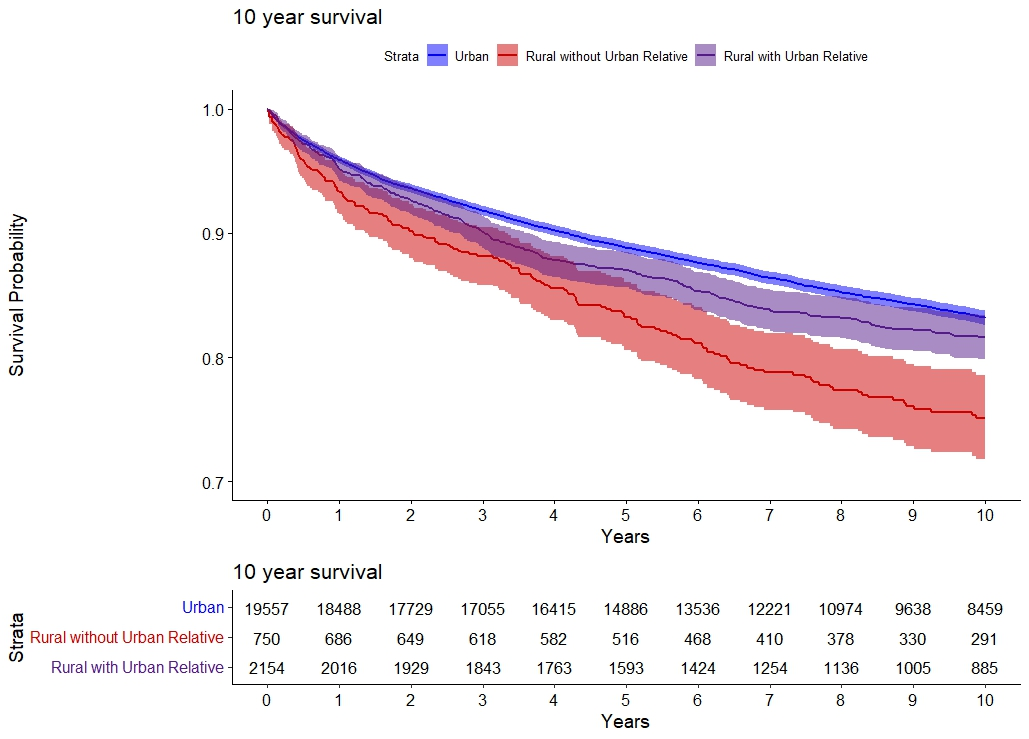


Supplementary Figure 2. Ten Year Cancer Specific Survival Curve
