# Supplementary material for: Urban relatives ameliorate survival disparities for genitourinary cancer in rural patients
Source: Cancer Med. 2024 Mar 13;13(5):e7058. doi: 10.1002/cam4.7058 (PMC10935886; doi:10.1002/cam4.7058)
Supplement: Supplementary file 2 — Table S1. Table S2. [file CAM4-13-e7058-s001.docx]

| **Supplemental Table 1:** Sensitivity analysis of 5 and 10 year overall survival of urban and rural groups, controlling for longevity of rural residence, when only examining first degree relatives, and when a minimum of 5 first degree relatives was required. | | | | | | |
| --- | --- | --- | --- | --- | --- | --- |
|  | Main Results | | Any First Degree Relatives | | Minimum 5 First Degree Relatives | |
|  | HR (95% CI) | p-value | HR (95% CI) | p-value | HR (95% CI) | p-value |
|  |  |  |  |  |  |  |
| **5 year OS** |  |  |  |  |  |  |
| Urban | REF |  | REF |  | REF |  |
| All Rural | 1.25 (1.14, 1.37) | <0.001 | 1.25 (1.14, 1.38) | <0.001 | 1.24 (1.12, 1.36) | <0.001 |
|  |  |  |  |  |  |  |
|  |  |  |  |  |  |  |
| Urban | REF |  | REF |  | REF |  |
| Rural without Urban Relative | 1.41 (1.22, 1.63) | <0.001 | 1.43 (1.22, 1.67) | <0.001 | 1.45 (1.23, 1.71) | <0.001 |
| Rural with Urban Relative | 1.19 (1.07, 1.32) | 0.002 | 1.19 (1.07, 1.33) | 0.001 | 1.17 (1.05, 1.31) | 0.006 |
| **10 year OS** |  |  |  |  |  |  |
| Urban | REF |  | REF |  | REF |  |
| All Rural | 1.22 (1.13, 1.31) | <0.001 | 1.21 (1.12, 1.31) | <0.001 | 1.21 (1.11, 1.31) | <0.001 |
| Urban | REF |  | REF |  | REF |  |
| Rural without Urban Relative | 1.46 (1.3, 1.65) | <0.001 | 1.45 (1.28, 1.66) | <0.001 | 1.5 (1.31, 1.71) | <0.001 |
| Rural with Urban Relative | 1.13 (1.04, 1.23) | 0.004 | 1.14 (1.05, 1.24) | 0.003 | 1.13 (1.03, 1.23) | 0.009 |
| Abbreviations: OS – overall survival, HR – Hazards Ratio | | | | | | |

| **Supplemental Table 2:** Sensitivity results of 5 and 10 year prostate-only and non-prostate genitourinary cancer survival. | | | | |
| --- | --- | --- | --- | --- |
|  | **5 Year OS** | | **10 Year OS** | |
|  | **HR (95% CI)** | **p-value** | **HR (95% CI)** | **p-value** |
| **Prostate Specific Survival** |  |  |  |  |
| Urban | REF |  | REF |  |
| Rural Without Urban Relative | 1.42 (1.16, 1.73) | <0.001 | 1.49 (1.28, 1.74) | <0.001 |
| Rural With Urban Relative | 1.2 (1.05, 1.38) | 0.009 | 1.16 (1.05, 1.29) | 0.005 |
| **Non-Prostate Survival** |  |  |  |  |
| Urban | REF |  | REF |  |
| Rural Without Urban Relative | 1.44 (1.16, 1.77) | <0.001 | 1.43 (1.18, 1.72) | <0.001 |
| Rural With Urban Relative | 1.18 (1, 1.39) | 0.052 | 1.08 (0.93, 1.26) | 0.287 |
| Abbreviations: OS – overall survival, HR – Hazards Ratio | | | | |
